# GAI Functions in the Plant Response to Dehydration Stress in *Arabidopsis thaliana*

**DOI:** 10.3390/ijms21030819

**Published:** 2020-01-27

**Authors:** Zhijuan Wang, Liu Liu, Chunhong Cheng, Ziyin Ren, Shimin Xu, Xia Li

**Affiliations:** 1 State Key Laboratory of Agricultural Microbiology, College of Plant Science and Technology, Huazhong Agricultural University, Wuhan 430070, China; wzhijuan@163.com (Z.W.); rzy1819345962@163.com (Z.R.);; 2 The State Key Laboratory of Plant Cell & Chromosome Engineering, Center for Agricultural Research Resources, Institute of Genetics and Developmental Biology, Chinese Academy of Sciences, 286 Huaizhong Road, Shijiazhuang 050021, China; liuliu19890729@163.com (L.L.); xiaobei15109217512@163.com (C.C.); 3 Graduate University of Chinese Academy of Sciences, No.19A Yuquan Road, Beijing 100049, China

**Keywords:** drought, GA, DELLA, ABF2, protein–protein interaction

## Abstract

DELLA (GAI/RGA/RGL1/RGL2/RGL3) proteins are key negative regulators in GA (gibberellin) signaling and are involved in regulating plant growth as a response to environmental stresses. It has been shown that the DELLA protein PROCERA (PRO) in tomato promotes drought tolerance, but its molecular mechanism remains unknown. Here, we showed that the *gai-1* (gibberellin insensitive 1) mutant (generated from the *gai*-*1* (L*er*) allele (with a 17 amino acid deletion within the DELLA domain of GAI) by backcrossing *gai*-*1* (L*er*) with Col-0 three times), the gain-of-function mutant of GAI (GA INSENSITIVE) in *Arabidopsis*, increases drought tolerance. The stomatal density of the *gai-1* mutant was increased but its stomatal aperture was decreased under abscisic acid (ABA) treatment conditions, suggesting that the drought tolerance of the *gai-1* mutant is a complex trait. We further tested the interactions between DELLA proteins and ABF2 (abscisic acid (ABA)-responsive element (ABRE)-binding transcription factors) and found that there was a strong interaction between DELLA proteins and ABF2. Our results provide new insight into DELLA proteins and their role in drought stress tolerance.

## 1. Introduction

A water deficit is a restrictive factor for plant development, productivity, and geographical distribution. Plants have evolved varied strategies to cope with decreased water availability, including promoting stomatal closure and altered plant growth and development. The stress-induced hormone abscisic acid (ABA) plays an important role in a plant’s response to drought tolerance [1,2,3]. Increasing evidence has proven that gibberellin (GA) plays a negative role in drought response. The over-accumulation of the GA mutant or increased GA activity shows an increased water deficit sensitivity, whereas a GA-deficient mutant or decreased GA activity shows an increased water deficit tolerance [4,5,6,7,8]. 

DELLA (GAI/RGA/RGL1/RGL2/RGL3) proteins are major negative regulators of GA signaling. In the absence of GA, DELLA proteins inhibit the GA-dependent processes, including germination, growth, and flowering. Under increased GA levels, GA binds to its nuclear receptor GID1 (GA insensitive dwarf1) and changes its conformation, leading to its interaction with the N-terminal end of DELLA proteins [9,10,11]. The interaction of DELLA proteins with GID1 causes its ubiquitination and subsequent degradation by the 26S proteasome, leading to the activation of GA responses [12,13]. DELLA proteins are involved in most GA-mediated plant growth and environmental stresses, including dehydration stress. Recently, it was reported that the DELLA protein PRO (PROCERA) in tomato functions positively in the plant response to drought stress. The loss-of-function of the *PRO* mutant shows a reduced tolerance to drought, whereas the overexpression of the constitutively active stable *PRO* increases drought tolerance [8]. However, the molecular mechanism of DELLA proteins remains unclear. 

There are five DELLA members in Arabidopsis: GAI (GA INSENSITIVE), RGA (REPRESSOR OR GAI3), RGL1 (RGA-LIKE1), RGL2, and RGL3. To uncover the molecular mechanism that determines how DELLA proteins function in drought tolerance, we used GAI as a sample to analyze the function of DELLA proteins in response to drought stress. We made use of a gain-of-function mutant *gai-1* generated from the *gai*-*1* (L*er*) allele (with a 17 amino acid deletion within the DELLA domain of GAI) by backcrossing *gai*-*1* (L*er*) with Col-0 (Columbia-0) three times. We showed that this mutant has an increased drought tolerance phenotype. Further, we found that GAI and other DELLA proteins interacted with ABF2 (abscisic acid (ABA)-responsive element (ABRE)-binding transcription factors), the transcriptional factor that plays a pivotal role in ABA signaling for drought tolerance. Our results thus shed some light on the mechanism behind how DELLA proteins function in drought stress tolerance.

## 2. Results

**Figure 1:**
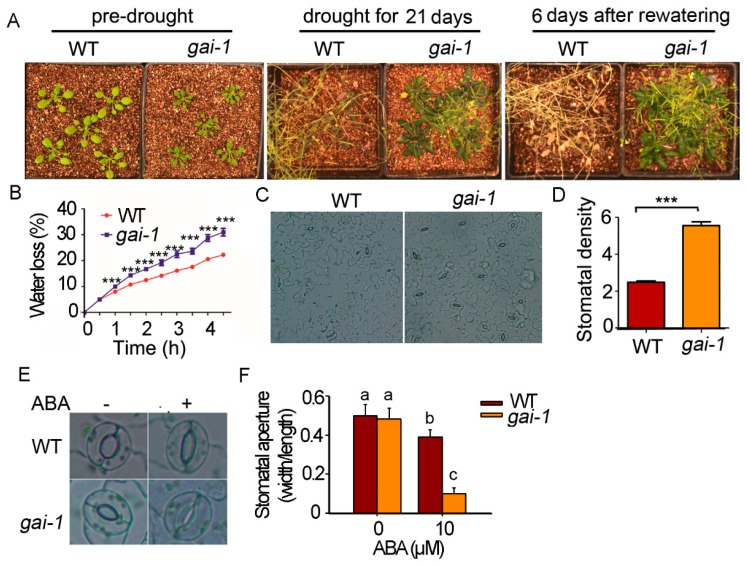
The *gai-1* (gibberellin insensitive 1) mutant is more tolerant to drought stress than WT (wild type). (**A**) *gai-1* mutant plants showed tolerance to dehydration stress. *gai-1* mutant plants showed the ability to withstand long drought conditions without negative effects whereas the wild type under the same conditions completely wilted. (**B**) *gai-1* plants showed increased water loss compared to WT. Data shown are the means ± SDs from three biological repeats (*n* = 3, eight leaves from eight plants were used for each repeat, *p* < 0.001). (**C**,**D**) Stomatal density of WT and *gai-1* mutant. Stomatal density was observed from comparable age leaves of 3 week old wild type and *gai-1* plants. The stomatal density was represent by number of stomata per millimeters squared. Data shown are the means ± SDs from three biological repeats (*n* = 3, five leaves from five plants were used for each repeat, *p* < 0.001). (**E**) Representative stomata of the WT and *gai-1* mutant under control and abscisic acid (ABA) treatment conditions. Leaves of the WT and *gai-1* mutant were treated with 10 μM ABA for 2 h (+), and (−) represents leaves without ABA treatment. (**F**) Stomatal apertures of the WT and *gai-1* mutant corresponding to (**E**). Values are mean ratios of width to length ± SDs of three independent experiments. Letters indicate significant differences from the WT (0 ABA treatment) according to the Student’s Newman–Kuels test (*** *p* < 0.05).

To study the function of DELLA proteins in drought tolerance in Arabidopsis, we firstly analyzed the phenotype of the *gai-1* mutant under the condition of drought treatment. Three-week-old seedlings of wild type (Col-0) and mutant *gai-1* were withheld from water for 21 days. The wild type plants were severely wilted, whereas the *gai-1* mutant did not wilt and continued to grow. After rewatering, all of the *gai-1* plants recovered, whereas none of the wild type plants survived (Figure 1A), indicating that the *gai-1* mutant is more tolerant to drought and that GAI is a positive regulator in the plant response to drought tolerance. The function of GAI in drought tolerance is consistent with that of PRO in tomato, suggesting that this tolerance is a conserved function of DELLA proteins in the plant kingdom. 

Changes in transpiration rate could account for the altered tolerance to drought. We then tested the water loss rate of the detached leaves. Leaves of 3 week old seedlings were cut and exposed to air and were weighted at regular time points. To our surprise, the *gai-1* mutant leaves lost their water at a much higher rate than the wild type leaves (Figure 1B). The water loss of the wild type sample was only 20%, whereas the water loss of the *gai-1* mutant was over 30% at 4 h after exposure to air, suggesting that the *gai-1* mutant is sensitive to dehydration when detached leaves are exposed to air. 

The stomata are key channels that control gas exchange and water evaporation. We then tested the stomatal density and aperture from leaves of wild type and *gai-1* plants grown in soil. The stomatal density of the *gai*-*1* mutant was significantly higher than that of the wild type plant (2.6×) (Figure 1C,D). This may be the reason for the higher rate water loss in the *gai-1* mutant for the detached leaves. For the stomatal apertures, the wild type and *gai-1* mutant were comparable under KCl-treated control conditions. However, under ABA treatment, the stomatal aperture of the *gai-1* mutant was much smaller than that of the wild type (Figure 1E,F). The stomatal density and aperture of the *gai-1* mutant under stress conditions are consistent with those of the *PRO* gain-of-function mutant, suggesting that this is a conserved mechanism for DELLA proteins in regulating plant development and environmental adaption.

**Figure 2:**
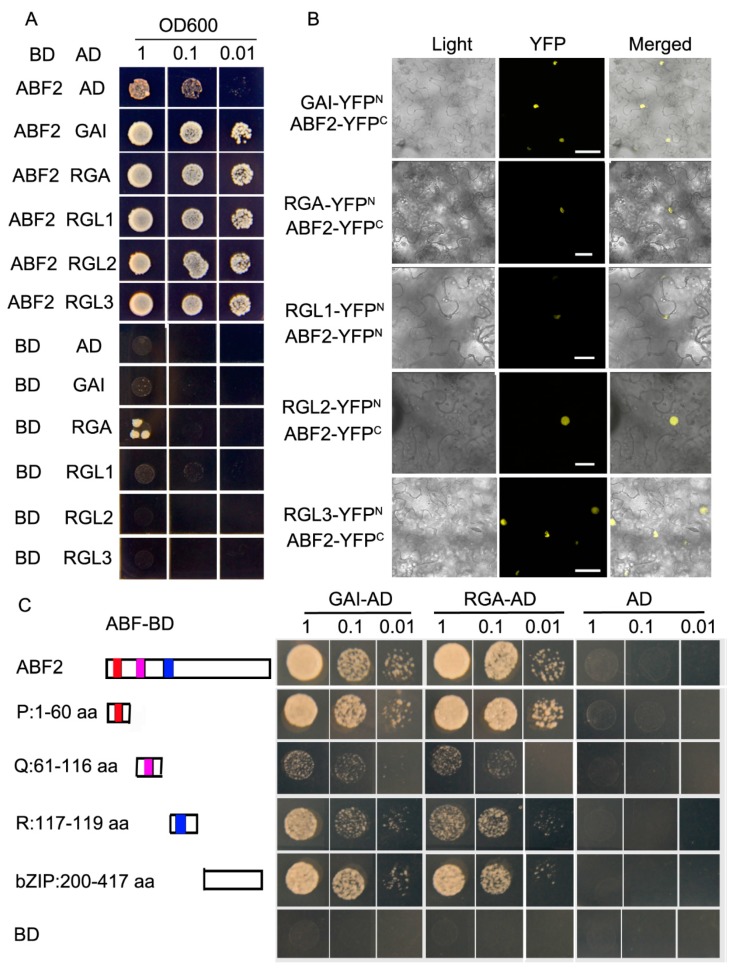
DELLA (GAI/RGA/RGL1/RGL2/RGL3) proteins interacted with ABF2 (abscisic acid (ABA)-responsive element (ABRE)-binding transcription factors). (**A**) DELLA proteins interacted with ABF2 in yeast two-hybrid assay. The yeast cells expressing the indicated constructs were spotted as a series of three dilutions. The yeast cells expressing the constructs of ABF2-pGBKT7-DEST (BD)/GAI-pGADT7-DEST (AD), ABF2-BD/RGA (REPRESSOR OR GAI3)-AD, ABF2-BD/RGL1 (RGA-LIKE1)-AD, ABF2-BD/RGL2-AD, and ABF2-BD/RGL3-AD grew better on the SD medium than that of yeast growth cells expressing the control’s constructs. (**B**) BiFC (bimolecular fluorescent complimentary) assay between DELLA proteins and ABF2. *Nicotiana benthamiana* leaves were co-transformed with the constructs containing the indicated YFP (yellow fluorescent protein) N-terminal (YFP^N^) and YFP C-terminal (YFP^C^) fusions, and YFP was imaged 48 h after transformation. Bars = 50 μm. (**C**) Interaction assay between GAI and RGA with ABF2 fragments. P: 1–60 amino acid; Q: 61–116 amino acid; R: 117–199 amino acid; bZIP (basic region/leucine zipper): 200–417 amino acid. The yeast cells expressing the indicated constructs were spotted as a series of three dilution. The yeast cell expressing the constructs of ABF2-BD/GAI-AD, ABF2-BD/RGA-AD, ABF2P-BD/GAI-AD, ABF2P-BD/RGA-AD, ABF2bZIP-BD/GAI-AD, ABF2bZIP-BD/RGA-AD, grew more effectively on the SD medium than that of yeast cells expressing ABF2R-BD/RGA-AD, ABF2R-BD/GAI-AD, ABF2Q-BD/ RGA-AD, and ABF2Q-BD/GAI-AD.

Generally, DELLA proteins function by interacting with other transcriptional factors. As ABF2 (abscisic acid (ABA)-responsive element (ABRE)-binding transcription factors) is a key regulator in drought tolerance, we hypothesized that GAI interacts with ABF2 to increase drought tolerance. To test this hypothesis, we tested the interaction between GAI and ABF2 via yeast two-hybrid and BiFC (bimolecular fluorescent complimentary) assays. In the yeast two-hybrid assay, GAI and ABF2 were recombined to the gateway-compatible destination vectors pGADT7-DEST (AD) and pGBKT7-DEST (BD), respectively. The AD and BD constructs were cotransformed to the yeast strain AH109, and their interaction was determined by the growth on the SD (synthetic dropout) medium lacking Trp (tryptophan), Leu (leucine), His (histidine), and Ade (adenine). There was a strong interaction between GAI and ABF2 in the yeast two-hybrid assay (Figure 2A). For the BiFC assay, GAI and ABF2 were recombined to pEarleyGate201-YN (N-terminal YFP (yellow fluorescent protein)) and pEarleyGate202-YC (C-terminal YFP), respectively. Both constructs were coinfiltrated into *Nicotiana benthamiana* leaves. The YFP signal was observed in the nucleus of the plant cell coexpressing GAI-YFP^N^ and ABF2-YFP^C^ (Figure 2B), but no YFP signal was detected in the plant cell coexpressing GAI-YFP^N^ and empty YFP^C^ or ABF2-YFP^C^ and empty YFP^N^ (Figure S1), indicating that GAI and ABF2 interacted in the nucleus of the plant cell. We also tested the interactions between other DELLA proteins, including RGA, RGL1, RGL2, and RGL3, with ABF2. The yeast two-hybrid and BiFC assays both showed that all of the DELLA proteins interacted with ABF2 (Figure 2A,B). 

There are three conserved motifs in the N-terminal of ABF2: P, Q, and R. The P motif is responsible for transactivation activity and activates downstream gene expression [14]. To test which motif is responsible for interacting with GAI, we tested the interaction between GAI and the P, Q, R, and bZIP (basic region/leucine zipper) motifs of ABF2. Our yeast two-hybrid assay showed that there was a strong interaction between the GAI and the P and bZIP motifs, whereas the interaction between GAI and the Q and R motifs was much weaker (Figure 2C). We also tested the interaction between RGA and the P, Q, R, and bZIP motifs of ABF2. RGA also showed a strong interaction with the P and bZIP motifs but a weak interaction with the Q and R motifs (Figure 2C). 

## 3. Discussion

GA is an important phytohormone that regulates plant growth. Increasing evidence has demonstrated that GA plays a role in the response to environmental stresses. DELLA proteins are key negative regulators in GA signaling, and our results showed that DELLA proteins increased drought tolerance by interacting with ABF2, a positive regulator in the ABA signaling pathway.

The cellular mechanism of the drought tolerance of the *gai-1* mutant seems complex and confusing. The stomatal density of the *gai-1* mutant is higher than that of the wild type, which makes water loss occur more quickly. Indeed, we found that the water loss rate of the detached leaves in the *gai-1* mutant was higher than that of the wild type (Figure 1B). The stomatal aperture in ABA treatment was found to be smaller in the *gai-1* mutant than that of the wild type sample, which may be responsible for the drought tolerance. The stomata’s phenotype, density, and aperture of the *gai-1* mutant are similar to those of the gain-of-function of *PRO* in tomato [8], suggesting that it is conserved for DELLA proteins in regulating plant development and stress response.

As DELLA proteins do not have a DNA binding domain, it is common for them to interact with other transcription factors to regulate downstream target genes. For example, DELLA proteins interact with ABI3 and ABI5 to activate *SOM* (*SOMNUS*) expression at high temperatures [15]; RGA interacts with BZR1 (Brassinazole-resistant 1) to inhibit its transcriptional activities to downstream genes to regulate cell growth [16]; RGA interacts with WRKY6 (WRKYGQK) to block its transcriptional activities on its downstream genes, *SAG13* (Senescence-associated gene13) and *SGR* (Stay green), to regulate senescence [17]; and RGL2 interacts with the NF-YC (NUCLEAR FACTOR-Y C) homologues NF-YC3, NF-YC4, and NF-YC9 to activate the downstream gene ABI5 to regulate seed germination [18]. Here, we showed that DELLA proteins interact with ABF2 to regulate drought tolerance. AREB/ABF (abscisic acid-responsive element binding) proteins play pivotal roles in the regulation of plant responses to abiotic stresses. By binding to the ABRE element in the promoter region of stress-responsive genes, AREB/ABF factors regulate gene expression under drought stress [19,20]. In Arabidopsis, four AREB/ABF factors, namely, AREB1/ABF2, AREB2/ABF4, ABF1, and ABF3, are induced by ABA and osmotic stress [21,22]. Overexpressing *AREB1*/*ABF2*, *AREB2*/*ABF4*, or *ABF3* promotes drought tolerance, and a loss-of-function of these genes enhances drought sensitivity [14,16,23,24]. Many stress-inducible genes, including *RD29B* and *RAB18*, were downregulated in the *areb1 areb2 abf3 abf1-2* quadruple mutant [20]. Our results showed a strong interaction between DELLA proteins and the ABF2 protein (Figure 2A,B). It is reasonable that the interaction of DELLA proteins with ABF2 could activate ABF2 transcriptional activity to promote drought tolerance. A further expression assay of the downstream gene in the *gai-1* mutant and the binding assay of ABF2 to the promoter of *RD29B* or *RAB18* in the presence or absence of DELLA proteins would allow for the determination of the role of the interaction between DELLA proteins and ABF2. We also cannot exclude the possibility that DELLA proteins interact with other AREB/ABF factors, such as AREB2/ABF4, ABF1, or ABF3. A further interaction assay between the DELLA proteins and other AREB/ABF factors will deepen our understanding of the role of DELLA proteins in drought tolerance.

In summary, our results showed that GAI increased drought tolerance in Arabidopsis. GAI had conserved functions in increasing the stomatal density and decreasing the stomatal aperture under ABA treatment conditions. Further, we showed that GAI interacted with ABF2, especially the N-terminal end P domain and the bZIP domain of ABF2. Our results provide new insight into DELLA protein functions in drought stress tolerance and the crosstalk between ABA and GA in response to drought tolerance.

## 4. Materials and Methods

### 4.1. Plant Materials and Growth Conditions

*Arabidopsis thaliana* ecotype Col-0 was used as the wild type in this study. The *gai-1* mutant was kindly gifted by Dr. Xiangdong Fu (Institute of Genetics and Developmental Biology, CAS). The seeds were germinated and grown on MS (Murashige & Skoog) medium and transplanted into soil at 10 days after germination. The plants were grown under a 16 h light/8 h dark photoperiod at 23 °C. 

### 4.2. Drought Treatment, Water Loss Analysis, and Stomatal Aperture Measurement

For measurement of drought tolerance, water was withheld from 21 day old wild-type and *gai-1* mutant plants. After 21 days of drought treatment, the plants were rewatered; the plants were photographed 6 days after re-watering. For measurement of water loss, eight rosette leaves from eight plants were detached from 3 week old well-watered plants and weighted at the indicated times. For the stomatal function, rosette leaves from well-watered plant were incubated in a solution containing 50 mM KCl, 10 mM CaCl_2_, and 10 mM MES (pH 6.15) for 2 h under light. ABA was then added to the solution to a final concentration of 10 μM. After ABA treatment for 2 h, stomatal apertures were measured as described previously [2].

### 4.3. Protein–Protein Analysis

The constructs were created in two pairs of Gateway-compatible destination vectors: pGBKT7-DEST (BD) with pGBAD7-DEST (AD) and pEarleyGate201-YN (N-terminal YFP) with pEarleyGate202-YC (C-terminal YFP) [25]. The coding sequences of *GAI*, *RGA*, *RGL1*, *RGL2*, *RGL3*, *ABF2*, and different deletion fragments of *ABF2* were amplified from Col-0 cDNA, inserted into pDONR207, and then recombined in the appropriate destination vector. Yeast two-hybrid and BiFC assays were performed as previously described [2]. For Y2H, *Saccharomyces cerevisiae* strain AH109 was used for co-transformation of the AD and BD constructs. A series of 5 μL aliquots of diluted co-transformed AH109 culture was spotted onto SD plates lacking Trp, Leu, His, and Ade, and incubated at 30 °C for 2–5 days. Plasmids pGBKT7 and pGADT7-Rec were used as negative controls. For the BiFC assay, *Agrobacterium tumefaciens* GV3101 carrying the YFP N-terminal and YFP C-terminal fusion constructs was infiltrated into *N. benthamiana* leaves, as described by Luo et al. [2]. The reconstituted YFP signals were observed using confocal imaging 48 h after infiltration. Empty vectors were used as negative controls. 

## Supplementary Materials

The following are available online at https://www.mdpi.com/1422-0067/21/3/819/s1.

## Abbreviations

ABA	Abscisic acid
AREB/ABF	Abscisic acid-responsive element binding
GA	Gibberellin
GAI	GA INSENSITIVE
GID1	GA insensitive dwarf1
PRO	PROCERA
