# Supplementary material for: GAI Functions in the Plant Response to Dehydration Stress in Arabidopsis thaliana
Source: Int J Mol Sci. 2020 Jan 27;21(3):819. doi: 10.3390/ijms21030819 (PMC7037545; doi:10.3390/ijms21030819)
Supplement: Supplementary file 1 [file ijms-21-00819-s001.pdf]

Supplemental Figure

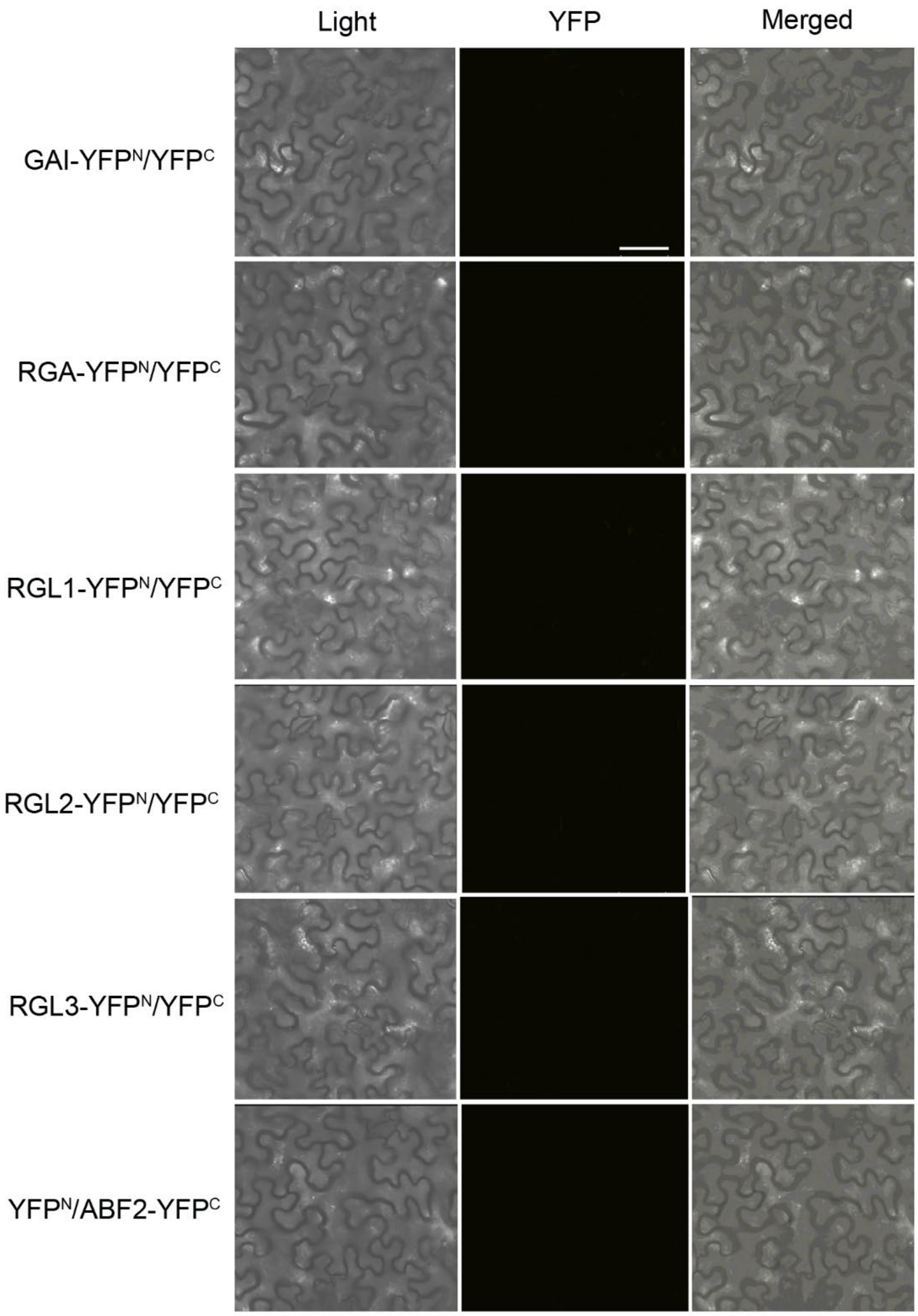

Figure S1. DELLA proteins interact with ABF2. Negative controls of BiFC assay between DELLA proteins and ABF2. N. benthamiana leaves were co-transformed with constructs containing the indicated YFP N-terminal (YFPN) and YFP C-terminal (YFPC) fusions, and YFP was imaged 48 h after transformation. Bar = 50 μm.
